# Common Factors of Meditation, Focusing, and Cognitive Behavioral Therapy: Longitudinal Relation of Self-Report Measures to Worry, Depressive, and Obsessive-Compulsive Symptoms Among Nonclinical Students

**DOI:** 10.1007/s12671-014-0296-0

**Published:** 2014-03-28

**Authors:** Tomoko Sugiura, Yoshinori Sugiura

**Affiliations:** 1 Japan Society for the Promotion of Science, Tokyo, Japan; 2 Graduate School of Integrated Arts and Sciences, Hiroshima University, 1-7-1, Kagamiyama, Higashi-Hiroshima City, Hiroshima Prefecture 739-8521 Japan

**Keywords:** Refraining from catastrophic thinking, Detached coping, Self-observation, Longitudinal design, Mediator, Metacognitions

## Abstract

Meditation has a long tradition with substantial implications for many psychotherapies. It has been postulated that meditation may cultivate therapeutic processes similar to various psychotherapies. A previous study used joint factor analysis to identify five common factors of items of scales purported to capture psychological states cultivated by meditation, focusing, and cognitive behavioral therapy, namely, refraining from catastrophic thinking, logical objectivity, self-observation, acceptance, and detached coping. The present study aimed to extend previous research on these five factors by examining their longitudinal relationship to symptoms of depression, obsession and compulsion, and worrying, with two correlational surveys without intervention. Potential mediators of their effect on worrying were also explored. Longitudinal questionnaire studies from two student samples (*n* = 157 and 232, respectively) found that (a) detached coping was inversely related to obsessive-compulsive symptoms about 5 weeks later; (b) detached coping was inversely related to depressive symptoms about 5 weeks later; (c) refraining from catastrophic thinking was inversely related to worrying, while self-observation was positively related to worrying about 2 months later; and (d) the relation of refraining from catastrophic thinking to worrying was mediated by negative beliefs about worrying, while the relation of self-observation to worrying was mediated by negative beliefs about worrying and monitoring of one’s cognitive processes. As refraining from catastrophic thinking involves being detached from one’s negative thinking and detached coping involves distancing oneself from external circumstances and problems, the results suggest that distancing attitudes are useful for long-term reduction of various psychological symptoms.

## Introduction

Meditation has a long tradition and significant implications for many clinical interventions. For example, Martin (1997, 2002) argued that mindfulness, an important characteristic of meditation, is also an important factor in all forms of psychotherapy. In addition, mindfulness meditation was recently incorporated into some forms of cognitive behavioral therapy (CBT) (e.g., Segal et al. 2002). For example, Teasdale et al. (2002) found that metacognitive awareness (or decentering; viewing one’s thoughts as mere mental phenomena rather than as reality) increased following CBT and mindfulness-based interventions when therapy was effective in reducing depression relapse. Other studies have also reported increased decentering in both CBT (Fresco et al. 2007) and mindfulness-based interventions (Carmody et al. 2009). In sum, meditation could cultivate the therapeutic factors common to various clinical interventions.

**Fig. 1:**
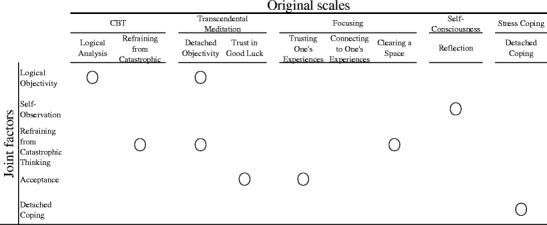
Correspondence of contents between the five joint factors and the original scales

Although intriguing as a theory, empirical support for Martin’s (1997, 2002) perspective is limited. Based on the reasoning that characteristics associated with meditation may be common factors with various psychotherapies, Y. Sugiura (2006) conducted a joint factor analysis of items from measures purported to capture psychological states cultivated by meditation and other interventions (CBT and focusing) together with other related variables (self-focus and stress coping). In a joint factor analysis, items from different measures are included in the same factor analysis. This method allows researchers to examine the possibility of factors that consist of items from different scales. The following five factors emerged: refraining from catastrophic thinking, logical objectivity, self-observation, acceptance, and detached coping. Some factors were derived not from the original scales but from combined items from different scales constructed based on different clinical interventions. The correspondence of contents between the original measures and obtained joint factors is demonstrated in Fig. 1. For example, refraining from catastrophic thinking includes items from scales based on CBT, focusing, and transcendental meditation, while logical objectivity combines items from scales based on CBT and transcendental meditation. The following paragraphs will explain each factor and subsequently report relations between the five common factors, depressive symptoms, and worrying to lay the foundation for the present study.

Refraining from catastrophic thinking reflects the skills necessary to detach from and to suspend negative thinking (e.g., “Even if bad consequences of a problem come to mind, I can reassure myself that they are nothing more than my imagination”). It resembles metacognitive awareness, or decentering, which constitutes perceiving negative thoughts as mere mental phenomena rather than as part of the self or reality (Fresco et al. 2007; Teasdale et al. 2002).

Self-observation (e.g., “I love exploring my “inner” self”) constitutes engagement in self-focus with curiosity and openness. Self-observation items are derived from the reflection scale (Trapnell and Campbell 1999), which was distinguished from maladaptive self-focus (rumination) in its relation to openness to experience. In fact, mindfulness is also related to openness (Baer et al. 2004; Bishop et al. 2004), and breathing meditation induces a curious attitude toward oneself.

Logical objectivity (e.g., “I can think of several alternatives about how to think or how to act”) resembles orthodox CBT skills, which emphasize active problem-solving. However, it also contains items from Transcendental Meditation. Indeed, logical objectivity and refraining from catastrophic thinking were positively correlated (*r* = .55; *p* < .001; Sugiura 2006). This is consistent with the finding that both standard CBT and mindfulness meditation enhance metacognitive awareness (e.g., Teasdale et al. 2002). Considered as a whole, logical objectivity seems to capture common factors of both CBT (or problem-solving) and meditation.

Detached coping (e.g., “Do not see the problem or situation as a threat”) is similar to refraining from catastrophic thinking in its emphasis on detachment and distancing, but differs in that its focus is external rather than internal. Wells and Matthews (1994) suggested that detached coping might capture clinically relevant decentering skills.

Acceptance (e.g., “Things always fall into place for me”; “I speak with confidence in my feelings”) relates to one’s feelings as well as the external environment. Importantly, this is different from acceptance defined as the inverse of experiential avoidance (aversion and avoidance of inner experiences) (Bond et al. 2011).

Sugiura (2006) correlated common factors with worrying and depressive symptoms and found that refraining from catastrophic thinking was the only significant negative predictor of depressive symptoms and one of the two negative predictors of worrying (the other was detached coping) after stepwise selection among the five common factors. Beta for refraining from catastrophic thinking on worrying was stronger than that for detached coping. Self-observation was unexpectedly positively related to both depressive symptoms and worrying, after stepwise selection.

Individual differences studies focusing on adaptive psychological states are important for elucidating the processes of psychotherapy and meditation. For example, D'Zurilla and Nezu (1990) developed the Social Problem-Solving Inventory (SPSI) to measure desirable problem-solving skills according to problem-solving therapy (D'Zurilla 1986). The development and the refinement of problem-solving therapy largely depended on studies that used the SPSI. For example, a factor analytic study with nonclinical students using the SPSI led to the reconceptualization of adaptive problem-solving skills (Maydeu-Olivares and D'Zurilla 1996). In addition, Frye and Goodman (2000) used the revised SPSI and found that adaptive problem-solving orientation moderated the effect of stressors on depression among adolescent girls. Individual differences in mindfulness have also been measured and yielded empirical findings outside the context of meditation intervention (e.g., Baer et al. 2006; Brown and Ryan 2003).

In addition, studies of individual differences in psychological symptoms among nonclinical samples are informative for understanding clinical intervention (analogue studies), such as Frye and Goodman’s study (2000) discussed above. Studies have revealed that depression, one of the symptoms correlated with meditation-related factors in Sugiura (2006), is continuous from clinical to nonclinical populations (Flett et al. 1997). This view was supported by evidence from a large community sample (Okumura et al. 2009).

Similarly, the attempt to elucidate common factors of meditation and some forms of psychotherapy by individual difference studies is an important line of research. However, the results of Sugiura (2006) were based on a cross-sectional design that included only worry and depression; the author did not consider possible mediators of the common factors, especially the known etiological factors for symptoms. This study sought to enhance the understanding of common meditation-related factors with a longitudinal design and broadened correlates. To this end, the following paragraphs will introduce obsessive-compulsive symptoms as an additional dependent variable and metacognitive beliefs as a potential mediator of worrying.

Obsessive-compulsive symptoms are not confined to patients with obsessive-compulsive disorder (OCD) but can be found among nonclinical populations (Clark and Rhyno 2005; Muris et al. 1997; Rachman and de Silva 1978). Based on the finding that people without a diagnosis of obsessive-compulsive disorder also experience intrusive thoughts similar in content to those reported by people with a diagnosis of obsessive-compulsive disorder (Rachman and de Silva 1978), Salkovskis (1985) proposed that cognitive appraisals of intrusions or their contents could influence the progression of normal intrusions into clinical obsessive-compulsive symptoms. As this model emphasizes negative reactions to inner experience in the etiology of OCD, mindfulness meditation may be effective for OCD.

Wilkinson-Tough et al. (2010) found that a mindfulness-based intervention reduced OCD symptoms in a case series (*N* = 3), suggesting that reduction in thought-action fusion (confusing the thoughts with actual behavior and one's morality) and thought suppression (counter-productive thought control strategy) are mediating factors. As these potential mediators represent over-involvement in one’s thoughts, a detached stance toward one’s thoughts may reduce OCD symptoms. Hanstede et al. (2008) demonstrated the effect of mindfulness in students with nonclinical obsessive-compulsive symptoms with a quasi-random design. The treatment effect was mediated by “letting go,” which is comparable to refraining from catastrophic thinking.

Worry is defined as a chain of negative, uncontrollable thoughts (Borkovec et al. 1983). It is a common symptom of many anxiety disorders, especially generalized anxiety disorder (GAD), a debilitating and difficult-to-treat anxiety disorder (Holaway et al. 2006). Pathological worrying without a diagnosis of GAD is a common phenomenon (Holaway et al. 2006). In addition, the continuity between nonclinical and clinical populations in the distribution of worry among large samples has been demonstrated empirically (Olatunji et al. 2010).

The metacognitive model of worrying and GAD by Wells (2000, 2006) suggests that both positive and negative beliefs about worry are involved in the development and maintenance of pathological worrying and GAD. Positive metacognitive beliefs center on the perceived utility of worrying and are thought to motivate worrying. On the other hand, negative metacognitive beliefs involve the perception of worrying as uncontrollable and dangerous. These are thought to exacerbate distress arising from worrying and motivate maladaptive thought-control strategies. Wells and Cartwright-Hatton (2004) developed a measure of metacognitive beliefs called the Meta-Cognitions Questionnaire Short Form based on Wells’ model. The questionnaire includes five dimensions: positive beliefs about worry (e.g., “Worrying helps me to solve problems”), negative beliefs about worry (e.g., “My worrying could make me go mad”), lack of cognitive confidence (e.g., “My memory can mislead me at times”), need to control thinking (e.g., “I should be in control of my thoughts all of the time”), and cognitive self-consciousness (e.g., “I am constantly aware of my thinking”). Negative beliefs have been demonstrated as a particularly strong predictor of worrying and GAD status, whereas positive beliefs appear to have relatively weak predictive power (e.g., de Bruin et al. 2005; Ruscio and Borkovec 2004; Sugiura 2007b; Wells and Carter 2001; Wells and Papageorgiou 1998).

In recent studies, mindfulness has successfully been applied to reduce GAD symptoms (Evans et al. 2008; Roemer et al. 2008). It is highly plausible that mindfulness, together with detachment from one’s internal experiences, works by reducing maladaptive metacognitions that represent excessive involvement with worrying (e.g., overestimation of the harm of worrying). Therefore, we included metacognitive beliefs measured by the Meta-Cognitions Questionnaire as potential mediators of the relation between the common five meditation-related factors (i.e., refraining from catastrophic thinking, logical objectivity, self-observation, acceptance, and detached coping) (Sugiura 2006) and worrying.

This study attempted to extend Sugiura’s (2006) study on the common meditation-related factors in the following three ways, which correspond to Sugiura’s (2006) limitations. First, we employed a longitudinal design to predict symptom change with a one- or two-month interval. By doing so, we would be better able to infer causality, in comparison to the cross-sectional design utilized by Y. Sugiura (2006). Second, obsessive-compulsive symptoms were added to worry and depression as additional target symptoms. As obsessive-compulsive symptoms are characterized by intrusive thoughts (obsessive thoughts), symptom reduction by refraining from catastrophic thinking, related to how one detaches from negative thoughts, is expected. In addition, because excessive monitoring of cognitive processes is implicated in the etiology of obsessive-compulsive symptoms (Janeck et al. 2003), we expected a positive relationship between obsessive-compulsive symptoms and self-observation. Third, we introduced known etiological cognitive factors for worry (namely, maladaptive metacognitive beliefs) as potential mediators of the effects of common factors on worry. Sugiura (2006) did not consider possible mediators such as known etiological factors of symptoms of interest.

This study utilized longitudinal surveys in two samples described in the Participants and Longitudinal Design sections. Participants from both samples completed symptom measures twice: Sample 1 participants completed measures of depressive symptoms and obsessive-compulsive symptoms 1 month apart, while sample 2 participants completed the worrying scale 2 months apart. Participants also completed questionnaires measuring five meditation-related factors that served as independent variables: 1 week before time 1 depressive symptoms and obsessive-compulsive symptoms in sample 1 and simultaneously with time 1 worrying in sample 2. Sample 2 participants also completed a scale for metacognitive beliefs at time 2.

First, we expected that meditation-related factors would differ in their relation to depressive symptoms, obsessive-compulsive symptoms, and worrying. In addition to Sugiura (2006), who found that refraining from catastrophic thinking negatively predicted depressive symptoms and worry, Sugiura and Sugiura (2013) found that it had negative cross-sectional relation to worrying. Furthermore, decentering, a construct similar to refraining from catastrophic thinking, has been found to be negatively related to depression and anxious arousal (Fresco et al. 2007) and social anxiety (Hayes-Skelton and Graham 2012), suggesting a relationship with a wide range of symptoms. Considering these findings, refraining from catastrophic thinking may predict later symptoms of depression and OCD (1 month and a week later) and worrying (2 months later). Detached coping may also predict reduced worrying 2 months later, as this was the other negative independent predictor of worry in Sugiura (2006). However, self-observation may be related to later exacerbated symptoms of depression and OCD (1 month and a week later) and worrying (2 months later), considering the repeated demonstrations of the difficulty of self-focus (Mor and Winquist 2002), its demonstrated cross-sectional relation with depressive symptoms and worrying (Sugiura 2006), and the findings of a relationship between excessive monitoring of cognitive processes and obsessive-compulsive disorder (Janeck et al. 2003).

Second, because worrying is characterized by excessive negative reactivity to intrusive cognitions (Wells 2000), the effect of refraining from catastrophic thinking on worry should be mediated by reduced negative metacognitive beliefs. We also explored any other possible mediations (e.g., other metacognitive dimensions also mediate the effect of refraining from catastrophic thinking on worrying, or some metacognitive dimensions will mediate the effect of other meditation-related predictors of worry, if any).

## Method

### Participants

There were two separate samples of students in this study. All participants were enrolled in introductory psychology courses and completed the questionnaires during class. Students were told that they could refuse to participate, although none refused. Sample 1 consisted of 157 students (85 men and 72 women) with a mean age of 19.50 years (SD = 1.77). Sample 2 consisted of 232 students (126 men, 104 women, and 2 unknown), with a mean age of 19.47 years (SD = 1.26).

### Longitudinal Design

Sample 1 participants completed meditation-related scales (time 0) and scales assessing depressive and obsessive-compulsive symptoms 1 week later (time 1). Approximately 1 month later (time 2), obsessive-compulsive and depression symptoms were again measured. Sample 1 data is part of a larger longitudinal study predicting obsessive-compulsive symptoms. The effects of obsessive beliefs and life events on obsessive-compulsive symptoms were reported in T. Sugiura and Sugiura (2009). Sample 2 participants completed meditation-related scales and worrying at time 1 and completed questionnaires assessing metacognitive beliefs and worry about 2 months later (time 2).

### Measures Related to Meditation

The five factors revealed in the joint factor analysis by Sugiura (2006) were used as meditation-related variables. The items were derived from the following scales. The correspondence of the contents between original scales and the resultant factors is demonstrated in Fig. 1.

#### Scale of Meditation-Related Cognitive Styles (SMCS; Sakairi 2004)

The SMCS comprises 24 items to measure the cognitive styles achieved during Transcendental Meditation (meditation with an emphasis on attentional concentration). This scale contains three subscales: detached objectivity, trust in good luck, and flexibility. Items were rated on a 5-point scale ranging from 1 (“not at all true”) to 5 (“very true”). As all items in the flexibility subscale were reverse-scored and the contents reflected worry, this subscale was not used as a guard against criterion contamination (confusion with symptoms); only detached objectivity and trust in good luck were used here. Detached objectivity has eight items (e.g., “I see things as they are” and “I can think objectively when I am in trouble”), and trust in good luck has five items (e.g., “Good things happen to me” and “Things always fall into place for me”). All three subscales had good internal consistency (*α* > .85); meditators scored higher than nonmeditators and students on all measures (Sakairi 2004). In addition, 10 weeks of Transcendental Meditation practice led to increases in subscale scores, except for trust in good luck (Sakairi 2004).

#### Cognitive Control Scale (CCS; Sugiura and Umaoka 2003)

The CCS was developed to measure the voluntary use of CBT-like skills in daily life. Items were constructed based on the cognitive techniques detailed in the CBT treatment manual (Freeman et al. 1990). This procedure suggests that CCS is expected to be a face-valid measurement. Participants were asked about their perceived ability to perform the tasks described in each item when anxious. Participants responded on a 4-point scale ranging from 1 (“I absolutely cannot”) to 4 (“I surely can”). This scale has two factorially derived subscales: logical analysis and refraining from catastrophic thinking. The former reflects active and objective problem-solving skills, while the latter measures the ability to detach from negative thinking in order to alleviate catastrophic cognitions. Logical analysis consists of six items, e.g., “I can think of several alternatives for how to think or act,” and refraining from catastrophic thinking comprises five items, e.g., “I don’t develop a bad scenario from the situation.” Both subscales of the CCS revealed good to acceptable internal consistency (*α* > .72; Sugiura et al. 2003; Sugiura and Umaoka 2003a, b). Furthermore, both subscales indicated convergent validity (Amari and Umaoka 2002; Sugiura 2007a, b; Sugiura et al. 2003).

Although the CCS is derived from CBT, meditation-based programs have been found to improve refraining from catastrophic thinking scores among outpatients with diverse mental disorders (pre-post *d* = 1.17; Katsukura et al. 2008) and analogue samples (pre-follow-up *d* = 1.15; Ito et al. 2009; and pre-post *d* = .90; Tanaka et al. 2011). In addition, Katsukura et al. (2008) found that increased refraining from catastrophic thinking scores were related to symptom reduction (*r* = −.87 to −.79), suggesting that refraining from catastrophic thinking may mediate therapeutic change in such interventions. These findings suggest that this measure could also be used to investigate meditation-related processes.

#### Focusing Manner Scale (FMS; Fukumori and Morikawa 2003)

The FMS was developed to measure focusing-like attitudes in daily life. This scale has 23 items rated on a 5-point scale, ranging from 1 (“not at all true”) to 5 (“very true”) in three subscales: trusting one’s experience (e.g., “I trust my inner feelings”), connecting to one’s experience (e.g., “I tend to pay gentle attention to inner experiences”), and clearing a space (e.g., “When faced with daily problems, I refrain from ruminating on them”). Alphas were good for total score (*α* = .83) and the three subscales (*α* > .70). Scores for the total FMS and two of the subscales (trusting one’s experience and clearing a space) were negatively correlated with psychological symptoms, as measured by the General Health Questionnaire (Goldberg 1978).

#### Rumination-Reflection Questionnaire (RRQ; Trapnell and Campbell 1999)

Trapnell and Campbell (1999) sought to distinguish adaptive from maladaptive self-focus. Reflection is adaptive (e.g., “I love exploring my “inner” self” and “I’m very self-inquisitive by nature”), while rumination is clearly a symptomatic dimension; thus, it was not included in the factor analysis (Sugiura 2006). The Japanese version of the scale translated by Takizaki and Koshikawa (2000) was used. The Japanese version indicated good internal consistency (*α* = .85) as well as convergent/divergent validity (Takizaki and Koshikawa 2000). The reflection subscale has six items (the number of items was reduced following a factor analysis of the Japanese version) rated on a 5-point scale from 1 (“not at all true”) to 5 (“very true”).

#### Coping Styles Questionnaire (CSQ; Roger et al. 1993)

The CSQ evaluates coping with four factors: rational coping, avoidance coping, emotional coping, and detached coping. Items are rated on a 4-point scale ranging from 1 (“never”) to 4 (“always”). Whereas three of these correspond to established coping dimensions (task-oriented, emotion-oriented, and avoidance-oriented; Endler and Parker 1990), detached coping is a new construct. Only ten items of the detached coping subscale were used in the joint factor analysis by Sugiura (2006). This subscale indicated good internal consistency (*α* = .90) and concurrent validity. Some sample items are, “See the situation for what it actually is and nothing more,” “Feel independent of the circumstances,” and “Respond neutrally to the problem.”

### Depression

#### Center for Epidemiological Studies Depression Scale (CES-D; Radloff 1977)

The CES-D is one of the most widely used depression scales, particularly in nonclinical populations. The Japanese version, which exhibits good psychometric properties, was developed by Yatomi et al. (1993) and contains 20 items. The CES-D was originally rated on a 4-point scale; however, the Japanese version requires respondents to rate each item in terms of how frequently they experienced such feelings on a 3-point scale ranging from 1 (“seldom”) to 3 (“frequently”). The CES-D revealed good internal consistency (time 1, *α* = .88; time 2, *α* = .89).

### Obsessive-Compulsive Symptoms

#### Obsessive-Compulsive Inventory-Revised (OCI-R; Foa et al. 2002)

The OCI measures distress due to a wide range of obsessive-compulsive symptoms. The revised version includes three items for each of the following symptoms: checking, hoarding, neutralizing, obsessing, ordering, and washing. A validation study by Foa et al. (2002) revealed that the OCI-R is useful for differentiating individuals with and without OCD. In addition, it also revealed good internal consistency and test-retest reliability. The OCI-R was carefully translated into Japanese, and the Japanese translation demonstrated good internal consistency in this study (time 1, *α* = .84; time 2, *α* = .86).

### Worrying and Metacognitive Beliefs

#### Penn State Worry Questionnaire (PSWQ; Meyer et al. 1990)

The PSWQ is a 16-item questionnaire with strong psychometric properties for measuring the frequency and intensity of worry (Startup and Erickson 2008). The Japanese version by Sugiura and Tanno (2000) exhibits psychometric properties comparable to those of the original version; it obtained good internal consistency (*α* = .92), test-retest reliability (*r* = .88), positive correlations with trait anxiety and depression, as well as discriminant validity as demonstrated by the formation of a distinct factor from obsessive symptoms among a student population (Sugiura and Tanno 2000).

#### Meta-Cognitions Questionnaire short form (MCQ-30; Wells and Cartwright-Hatton 2004)

The MCQ-30 is the 30-item abbreviated version of the full MCQ (Cartwright-Hatton and Wells 1997). This scale measures beliefs about worry and intrusive thoughts, with five subscales noted in the Introduction. Items are rated on a 4-point scale from 1 (“do not agree”) to 4 (“agree very much”). Higher scores reflect the existence of maladaptive metacognitive beliefs. T. Sugiura et al. (2003) translated the full MCQ into Japanese and obtained good reliability for each subscale. There are established psychometric properties for five subscales. Sugiura and Sugiura (2013) reported adequate internal consistencies for five subscales (*α* = .74–.88). All five subscales were correlated with diverse emotional disturbances including worrying, trait anxiety, and obsessive-compulsive symptoms (Wells and Cartwright-Hatton 2004). Sugiura (2007b) and Sugiura and Sugiura (2013) found that both positive and negative metacognitive beliefs were positively correlated with worrying, with the latter indicating incremental validity over many other predictors in predicting worrying.

## Results

Tables 1 and 2 display the summarized descriptive statistics and alpha reliabilities for samples 1 and 2, respectively. The obtained alphas were .74–.89 for sample 1 and .74–.89 for sample 2, indicating satisfactory to excellent reliability, which is especially important for the “reconstructed” subscales for meditation-related items. There was an overall decrease in obsessive-compulsive symptoms across 1 month in sample 1 (*t* = −3.76; *p* < 001), and worrying decreased across 2 months in sample 2 (*t* = −4.37; *p* < .001).

**Table 1:** Descriptive statistics of sample 1 (*n* = 157)

	Mean	*SD*	*α* value
Meditation-related factors time 0			
Logical objectivity	31.60	5.47	.83
Self-observation	27.06	7.42	.86
Refraining from catastrophic thinking	35.04	7.43	.84
Acceptance	25.74	5.59	.74
Detached coping	16.70	4.75	.83
Symptoms time 1 (1 week after time 0)			
Depressive symptoms	35.36	7.96	.88
Obsessive-compulsive symptoms	21.27	10.38	.86
Symptoms time 2 (1 month after time 1)			
Depressive symptoms	34.99	7.94	.89
Obsessive-compulsive symptoms	19.16	10.56	.87

**Table 2:** Descriptive statistics of sample 2 (*n* = 232)

	Mean	*SD*	*α* value
Meditation-related factors time 1			
Logical objectivity	31.98	4.96	.82
Self-observation	26.35	7.36	.87
Refraining from catastrophic thinking	35.97	7.07	.84
Acceptance	27.64	5.50	.76
Detached coping	17.98	3.94	.74
Metacognitive beliefs time 2 (2 months after time 1)			
Need to control	10.41	3.45	.74
Positive beliefs	12.32	3.50	.83
Negative beliefs	12.22	3.79	.81
Cognitive self-consciousness	12.56	4.21	.88
Cognitive confidence	12.71	4.24	.81
Worrying time 1	51.06	11.22	.89
Worrying time 2	48.45	11.09	.88

### Zero-Order Correlations

Tables 3 and 4 present zero-order correlations among study variables from sample 1 and sample 2, respectively. Consistent with Sugiura (2006), most of the five meditation-related scales were positively intercorrelated in both samples, while there was no correlation between self-observation and refraining from catastrophic thinking/detached coping in sample 2. Significant correlations ranged from weak to strong (sample 1, *r* = .18–.71, *p* < .05; sample 2, *r* = .20–.65, *p* < .01).

**Table 3:** Correlation among sample 1 variables (*n* = 157)

	Logical objectivity	Self-observation	Refraining from catastrophic thinking	Acceptance	Detached coping	CES-D time 1	CES-D time 2	OCI-R time 1	OCI-R time 2
Meditation-related factors time 0									
Logical objectivity	1.00	.38***	.61***	.41***	.60***	−.36***	−.38***	−.12	−.13
Self-observation		1.00	.18*	.29***	.26***	.06	.05	.23**	.21**
Refraining from catastrophic thinking			1.00	.57***	.71***	−.45***	−.44***	−.26***	−.25**
Acceptance				1.00	.43***	−.38***	−.37***	−.15****	−.13
Detached coping					1.00	−.34***	−.37***	−.02	−.12
CES-D time 1						1.00	.76***	.30***	.31***
CES-D time 2							1.00	.33***	.42***
OCI-R time 1								1.00	.84***
OCI-R time 2									1.00

*CES-D* Center for Epidemiological Studies Depression Scale, *OCI-R* Obsessive Compulsive Inventory-Revised

*p < .05; **p < .01; ***p < .001; ****p < .10

**Table 4:** Correlation among sample 2 variables (*n* = 232)

	Logical objectivity	Self-observation	Refraining from catastrophic thinking	Acceptance	Detached coping	Need to control	Positive beliefs	Negative beliefs	Cognitive self-consciousness	Cognitive confidence	Worrying time 1	Worrying time 2
Meditation-related factors time 1												
Logical objectivity	1.00	.29***	.63***	.43***	.65***	−.06	.16*	−.24***	.13*	−.15*	−.33***	−.23**
Self-observation		1.00	.08	.20**	.09	.17*	.15*	.22***	.47***	.16*	.18**	.22***
Refraining from catastrophic thinking			1.00	.47***	.60***	−.22***	.02	−.45***	−.15*	−.19**	−.57***	−.48***
Acceptance				1.00	.31***	−.07	.05	−.14*	−.02	−.27***	−.20**	−.11****
Detached coping					1.00	−.02	.12****	−.27***	.04	−.17**	−.42***	−.34***
Metacognitive beliefs time 2												
Need to control						1.00	.50***	.53***	.56***	.29***	.25***	.41***
Positive beliefs							1.00	.18**	.38***	.18**	.04	.18**
Negative beliefs								1.00	.46***	.39***	.59***	.74***
Cognitive self-consciousness									1.00	.26***	.28***	.43***
Cognitive confidence										1.00	.26***	.32***
Worrying time 1											1.00	.68***
Worrying time 2												1.00

*p < .05; **p < .01; ***p < .001; ****p < .10

In sample 1, refraining from catastrophic thinking was negatively correlated with both obsessive-compulsive and depression symptoms. Logical objectivity, acceptance, and detached coping inversely correlated with depressive symptoms. Self-observation positively correlated with obsessive-compulsive symptoms.

In sample 2, several negative correlations emerged between meditation-related measures and metacognitive beliefs/worrying. Refraining from catastrophic thinking was strongly correlated with negative metacognitive beliefs (*r* = −.45, *p* < .001) and worrying (*r* = −.48 to −.57, *p* < .001) at both time points. On the other hand, self-observation was positively related to metacognitive beliefs and worrying (*r* = .15–.47, *p* < .05) and particularly to cognitive self-consciousness (*r* = .47, *p* < .001). Except for positive beliefs, metacognitive beliefs were positively correlated with worrying at both times; positive beliefs were related to worrying only at time 2. Negative beliefs about worrying had a particularly strong relationship with worrying at both time points (*r* = .59–.74, *p* < .001).

### Longitudinal Prediction of Symptoms

In sample 1, two hierarchical regressions were conducted with depression and obsessive-compulsive symptoms as dependent variables, while one hierarchical regression was conducted in sample 2 with worrying as a dependent variable. In all three regression analyses, time 2 symptoms (depression, obsessive-compulsive symptoms, and worrying) served as dependent variables. The corresponding time 1 symptom (e.g., time 1 depression when the dependent variable was time 2 depression) was entered in step 1 as a covariate. In step 2, the five meditation-related factors (i.e., refraining from catastrophic thinking, logical objectivity, self-observation, acceptance, and detached coping) were entered and were selected by using stepwise selection (*p* < .05 for entry in the regression equation and *p* > .10 for removal). Stepwise selection was used because predictive power was expected to differ across meditation-related factors.

#### Sample 1: Depressive and Obsessive-Compulsive Symptoms (*n* = 157)

Time 1 depressive symptoms (1 month prior) significantly predicted depressive symptoms in time 2 (*β* = .71; *p* < .001), explaining 57 % of the variance (Table 5). Detached coping, measured 1 week before time 1 symptoms, was selected among the five meditation-related factors and explained a further 1 % of the variance of time 2 depressive symptoms (*β* = −.12; *p* < .05), indicating that detached coping measured 1 week before time 1 symptoms was inversely related to depressive symptoms about 1 month and 1 week later.

The presence of obsessive-compulsive symptoms at time 1 significantly predicted these symptoms at time 2 (*β* = .84; *p* < .001), explaining 71 % of the variance (Table 5). Again, detached coping was selected among the five meditation-related factors and explained a further 1 % of the variance (*β* = −.10; *p* < .05), indicating that detached coping was inversely related to obsessive-compulsive symptoms about 1 month and 1 week later.

**Table 5:** Stepwise regression analysis predicting depressive and obsessive-compulsive symptoms by meditation-related factors (*n* = 157)

Steps	Predictors	CES-D		OCI-R	
		Δ*R* ^2^	*β* value^a^	Δ*R* ^2^	*β* value^a^
1	CES-D time 1	.57***	.71***	–	–
	OCI-R time 1	–	–	.71***	.84***
2	Meditation-related factors time 0				
	Logical objectivity	.01*		.01*	
	Self-observation				
	Refraining from catastrophic thinking				
	Acceptance				
	Detached coping		−.12*		−.10*

*CES-D* Center for Epidemiological Studies Depression Scale, *OCI-R* Obsessive-Compulsive Inventory-Revised

*p < .05; **p < .01; ***p < .001

^a^Standardized regression coefficients are presented. Predictors in step 2 were selected by stepwise procedure

#### Sample 2: Worrying (*n* = 232)

Time 1 worrying symptoms significantly predicted 46 % of variance (*β* = .56; *p* < .001) in time 2 worrying symptoms (2 months later). In addition, self-observation (*β* = .14; *p* < .01) and refraining from catastrophic thinking (*β* = −.17; *p* < .01) together explained a further 3 % of the variance. This suggests that refraining from catastrophic thinking at time 1 was associated with reduced worrying about 2 months later while self-observation was associated with increased worrying.

### Mediation of the Effect of Common Factors on Worrying by Metacognitive Beliefs (Sample 2)

The above analyses indicated that refraining from catastrophic thinking was negatively related to later worrying, while self-observation was related to later exacerbated worrying. The final question is whether these effects of meditation-related factors on worrying were mediated by the metacognitive beliefs implicated in the etiology of worrying (Wells 2000). Refraining from catastrophic thinking represents how one deals with cognitions. Therefore, it is highly plausible that the effect of meditation-related factors was mediated by metacognitive beliefs. In addition, because both self-observation and MCQ cognitive self-consciousness include the scrutiny of inner experience, the effect of the former may be mediated by the latter.

Statistically speaking, mediation occurs when the indirect effect of an independent variable on a dependent variable is significant as a function of some other variable, the mediator (Preacher and Hayes 2008). To estimate the indirect effects through potential mediators (i.e., metacognitive beliefs), we followed Mallinckrodt et al.’s (2006) recommendation to use bootstrap estimation. The SPSS macro for bootstrap estimation was provided by Preacher and Hayes (2008). Before testing indirect effects, we selected potential mediators of the effect of meditation-related factors on time 2 worrying using a hierarchical regression with stepwise selection (*p* < .05 for entry in the regression equation and *p* > .10 for removal), predicting worrying by five MCQ-30 subscales after controlling for time 1 worrying. Negative beliefs about worrying (*β* = . 47; *p* < .001) and cognitive self-consciousness (*β* = .11; *p* < .05) were significant predictors of time 2 worrying after controlling for time 1 worrying and stepwise selection from the five MCQ dimensions (Δ*R* ^2^ = .19; *p* < .001). Therefore, these two metacognitive beliefs, with unique predictive relation to worrying, were candidate mediators.

First, the indirect effects of refraining from catastrophic thinking through negative beliefs about worrying and/or cognitive self-consciousness were examined while controlling for self-observation and time 1 worrying as covariates. The indirect pathway through negative beliefs was significant (*b* = −.15, SE = .06; 95 % bootstrap CI [−.29, −.05]), but not through cognitive self-consciousness (*b* = −.02, SE = .02; 95 % bootstrap CI [−.07, .00]). The direct effect of refraining from catastrophic thinking (again controlling for self-observation and time 1 worrying) was not significant (*b* = −.10, SE = .08, 95 % CI [−.26, .05]), indicating that the effect of this meditation-related dimension is perfectly mediated by negative metacognitive beliefs.

Second, the indirect effect of self-observation through negative beliefs, while controlling for refraining from catastrophic thinking and time 1 worrying as covariates, was significant (*b* = .11, SE = .04; 95 % bootstrap CI [.04, .21]). The indirect effect through cognitive self-consciousness was also significant (*b* = .08, SE = .04; 95 % bootstrap CI [.01, .16]). However, the direct effect of self-observation (again controlling for refraining from catastrophic thinking and time 1 worrying) was not significant (*b* = .02, SE = .07; 95 % CI [−.12, .16]), indicating that the effect of this meditation-related dimension is perfectly mediated by negative metacognitive beliefs and cognitive self-consciousness.

## Discussion

This study examined the longitudinal predictive power of factors related to meditation, focusing, and CBT on obsessive-compulsive symptoms, depressive symptoms, and worrying. In addition, we explored possible mediators of the effect of meditation-related factors on worrying. We found that (a) detached coping predicted reduced obsessive-compulsive symptoms about 1 month and 1 week later; (b) detached coping predicted reduced depressive symptoms about 1 month and 1 week later; (c) refraining from catastrophic thinking predicted reduced worrying about 2 months later, while self-observation predicted exacerbated worrying; and (d) the effect of refraining from catastrophic thinking on worrying was mediated by negative beliefs about worrying, while the effect of self-observation was mediated by both negative beliefs about worrying and cognitive self-consciousness.

Detached coping and refraining from catastrophic thinking were related to reduced symptoms. Each of these factors requires maintaining distance from upsetting stimuli and refraining from preoccupation with such stimuli. However, the former focuses on external events, whereas the latter focuses on internal experience. Although obsessive-compulsive symptoms include persistent and distressing internal experience (obsessions), they often concern circumscribed external problems (e.g., “Have I locked my door? If not, thieves could enter”). In addition, the symptoms are induced by external stressors in some depressive individuals (Hayley et al. 2005; Ma and Teasdale 2004). Such characteristics of depressive and obsessive-compulsive symptoms may explain their present longitudinal relation with detached coping.

Refraining from catastrophic thinking involves detached awareness of one’s distressing thoughts. Although the concerns of worriers are primarily centered on realistic daily problems as opposed to obsessions (Turner et al. 1992; Wells and Morrison 1994), their contents are often changing. This is why their pathological manifestation is called “generalized” anxiety disorder, which implies worry over nearly everything, that is, “worry after worry” (Roemer et al. 1997). Borkovec and Roemer (1995) found that GAD patients tend to worry about minor things in order to avoid thinking about more distressing thoughts. Therefore, in spite of the seemingly “realistic” nature of worrisome thoughts, the contents tend not to matter for that person; the manner in which people appraise and control their thoughts (i.e., metacognitions) is more important. Such characteristics of worrying are consistent with the finding that refraining from catastrophic thinking is the only significant negative longitudinal predictor of worrying. Among the underlying etiological factors of worrying, negative beliefs about the uncontrollability and danger of worrying have been found to be particularly strong predictors (Sugiura 2007a, b; Wells 2006), and refraining from catastrophic thinking seems to be antithetical to such negative metacognitions (Wells 2005; Sugiura and Sugiura 2013). Consistent with this reasoning, refraining from catastrophic thinking predicted later reduced worrying, an effect entirely mediated by negative metacognitive beliefs.

Self-observation was positively related to worrying. Although this dimension is distinct from maladaptive self-focus (rumination; Trapnell and Campbell 1999), Sugiura (2006) found that it was positively related to both depressive symptoms and worrying by using a cross-sectional design. Such findings elucidate the problematic outcomes associated with self-observation, even when it is related to openness. This study has provided an explanation for such problematic outcomes of self-observation by revealing the mediators of its association with worrying. No direct effect on worrying was observed, indicating that the two mediators fully explained the effect. Self-observation was positively related to negative beliefs about worrying, suggesting that analysis of inner processes tends to be evaluative, although it is also associated with open attitudes. In addition, self-observation was more strongly related to cognitive self-consciousness than to negative metacognitive beliefs (*β* = .45, *p* < . 001, after controlling for time 1 worrying and refraining from catastrophic thinking, while a corresponding beta for self-observation on negative beliefs was *β* = .15, *p* < . 01), a reasonable finding given the conceptual similarity. Although cognitive self-consciousness has been correlated with both obsessive-compulsive symptoms and worrying (Wells and Cartwright-Hatton 2004), a recent study indicated that it was uniquely related to obsessive thoughts rather than worrying (de Bruin et al. 2007). In addition, Sica et al. (2007) conducted a longitudinal study and found that cognitive self-consciousness was prospectively related to adaptive coping. Therefore, it appears that self-observation has a dual effect. Consequently, an explanation of its detrimental effect on worrying is evasive. However, it may be natural to assume that cognitive self-consciousness leads to heightened awareness of negative thoughts, which then amplifies associated distress and impairs otherwise successful problem-solving by occupying limited attentional resources (Mohlman 2009).

### Limitations and Future Directions

Before concluding, limitations and future directions must be considered. First, because we only tested students, an examination of a more diverse population is desirable. This concern is relevant to the present research problem because mindfulness, a construct closely related to the present factors, has been associated with increased education (Baer et al. 2008). Considering worrying (and other intrusive thoughts) as transdiagnostic phenomena (Kertz et al. 2012), the present finding may be applied to wider symptoms. However, a more specific examination of the relationship to each symptom is also necessary.

Second, the maladaptive effect of self-observation is a promising avenue for future research. As many psychotherapies and meditations involve the exploration of inner experience, it is premature to disregard self-observation; searching for possible moderators may be useful. Baer et al. (2008) found that meditation enhanced the adaptive effect of observation of experiences. In addition, Takata et al. (2012) found that attentional functioning moderated the relationship between observation of experiences and well-being.

Third, because metacognitive beliefs and worrying were measured simultaneously, our design prevented causal inference (Kazdin 2007). However, as the longitudinal effects of negative metacognitive beliefs on anxiety and depression 6 months later (Yilmaz et al. 2011) and worrying 4 months later (Sica et al. 2007) have already been demonstrated, a pathway from negative metacognitive beliefs to worrying can be assumed based on prior research. Future studies will enhance inference about causality by measuring mediators (metacognitive beliefs) before measuring the dependent variables (e.g., worrying).

Finally, the temporal dynamics of change in symptoms need consideration because participants did not receive an intervention. There was an overall decrease in obsessive-compulsive symptoms across 1 month in sample 1, and worrying decreased across 2 months in sample 2. One plausible explanation for these decreases is “meaning change” induced by the exposure to symptom items (Knowles et al. 1996). Knowles et al. found a decrease in anxiety scores from test to retest and a decrease for later items even within a test. They found that the experience of answering items changed the understanding of the constructs and meaning of single items in light of those constructs. However, while this mechanism may explain the overall decrease in symptoms, it may not cogently explain the effect of meditation-related factors. The “meaning change” explanation assumes that exposure to negative items makes the negative meanings more salient in later items. This is considered a priming effect, to which mindful individuals are less susceptible (Radel et al. 2009). In fact, refraining from catastrophic thinking reflects being less influenced by negative cognitions. Therefore, the function of refraining from catastrophic thinking (and perhaps detached coping) may be antithetical to meaning change. Another explanation for the overall decrease in symptoms is cohort effects, which are also unlikely. Sample 1 participants completed the measures in mid-November and mid-December, and there were no specifiable events common to students (e.g., end-of-term examinations or a vacation break) identified between the two measurement times. Sample 2 completed the measures in mid-November and mid-January, on either side of the New Year’s break, with end-of-term examinations scheduled for the end of January. In spite of the difference in the timing of the measurements, both samples evidenced a decrease in symptoms (obsessive-compulsive symptoms or worry). Furthermore, the participants were thought to be well beyond the college newcomer's adaptation period because university terms in Japan start in April.

It is possible that meditation-related factors led to a decrease in symptoms in a certain subgroup of participants. Even in this case, changes in symptoms in that subgroup may be reflected in trends in the full sample. For example, meditation-related factors might be related to decreased symptoms in those with higher or lower symptoms at time 1. However, when the interaction terms between time 1 symptoms and meditation-related factors were added to regressions (Tables 5 and 6), these did not add to the predictive power (Δ*R* < .00; *p* > .20). Another probable explanation is that each participant was affected by internal factors, not linked to a life event, which influenced symptoms. For example, depression can relapse without the occurrence of particular life events, and mindfulness-based intervention was found to be effective for preventing relapses not instigated by life events (Ma and Teasdale 2004). An extension from such findings is that meditation-related factors, especially refraining from catastrophic thinking, can help one deal with mood deterioration instigated by internal factors. In the future, more than two measurement times will strengthen any inferences about causality. Nevertheless, longitudinal surveys based on two time points are common and have been fruitfully utilized for the prediction of later symptoms (Kivimaki et al. 2000; Selfhout et al. 2008).

**Table 6:** Stepwise regression analysis predicting worrying by meditation-related factors (*n* = 232)

Steps	Predictors	Δ*R* ^2^	*β* value^a^
1	Worrying time 1	.46***	.56***
2	Meditation-related factors time 1		
	Logical objectivity	.03***	
	Self-observation		.14**
	Refraining from catastrophic thinking		−.17**
	Acceptance		
	Detached coping		

**p < .01; ***p < .001

^a^Standardized regression coefficients are shown. Predictors in step 2 were selected by stepwise procedure

## Conclusions

In conclusion, this paper indicated that two facets of meditation-related factors were inversely related to later psychological symptoms. The effect of refraining from catastrophic thinking, which combines items from scales based on meditation, CBT, and focusing, suggests that the common mechanism underlying these diverse interventions is maintaining distance from one’s experiences. This is consistent with the now widely shared notion that how one relates to one’s experience determines adaptive outcomes (e.g., Hayes et al. 1999; Wells 2005). However, this study found that not only relation to inner experience but also relation to external environments (detached coping) affects one’s psychological symptoms, although maintaining distance remains important. As one recent study found a correlation between a measure of mindfulness in Langer's (1989) conception and those from the Buddhist tradition (Pirson et al. 2012), awareness of both internal and external events may constitute a promising focus of future research.

This study employed a longitudinal design; thus, a stronger causal inference is possible compared to the cross-sectional data in Sugiura (2006). Determining causality is of utmost consequence because Sugiura (2006) found a negative correlation between neuroticism and all of the meditation-related factors except self-observation. As neuroticism is a long-lasting trait factor, it is reasonable to assume that dispositional emotional distress played a role in reducing meditation-related factors. The results of this study suggest that, despite the prohibiting influence of trait negative emotions, detached attitudes affect later psychological distress.
